# Effect of elevated irrigation bottle height during cataract surgery on corneal endothelial cells in porcine eyes

**DOI:** 10.1186/s12886-023-02954-w

**Published:** 2023-05-11

**Authors:** Daniel A. Wenzel, Constanze Schultheiss, Vasyl Druchkiv, Olaf J. C. Hellwinkel, Martin S. Spitzer, Maximilian Schultheiss, Maria Casagrande, Nils Alexander Steinhorst

**Affiliations:** 1 University Eye Hospital, Centre for Ophthalmology, University Hospital Tübingen, Tübingen, Germany; 2 Department of Ophthalmology, University Medical Center Hamburg-Eppendorf, Hamburg, Germany; 3 Department of Obstetrics and Fetal Medicine, University Medical Center Hamburg-Eppendorf, Hamburg, Germany; 4 Department of Forensic Medicine, University Medical Center Hamburg-Eppendorf, Hamburg, Germany

**Keywords:** Irrigation, Aspiration, Bottle height, Corneal endothelium, Cataract, Cataract surgery, Phacoemulsification

## Abstract

**Background:**

Cataract surgery induces corneal endothelial cell loss (ECL). This study investigates the relationship between bottle height (BH) and ECL induced due to irrigation and aspiration (I/A) in cataract surgery and quantifies protective effects of intraoperatively used ophthalmic viscoelastic substances.

**Methods:**

Intermittent I/A without phacoemulsification was performed in porcine eyes for 10 min with varying BHs of 100 cm (BH100), 125 cm (BH125), 150 cm (BH150) or no treatment (control, no I/A). Additionally, in one group a dispersive ophthalmic viscoelastic substance was injected into the anterior eye chamber before treatment with I/A at a BH of 150 cm (BH150 + V). After exposure of the corneal endothelium to I/A, the corneas were prepared to split corneal buttons on day 0 and cultivated for 15 days. Endothelial cell density (ECD) was analyzed blinded on days 1, 8 and 15.

**Results:**

Relative ECL significantly correlated with irrigation BH (control (*n* = 13): -9.69 ± 6.03% (average ± standard deviation); BH100 (*n* = 12): -9.69 ± 4.81%—*p* = 1.000; BH125 (*n* = 14): -19.44 ± 7.30% – *p* < 0.001; BH150 (*n* = 13): -21.99 ± 6.70%—*p* < 0.001). I/A-induced ECL was significantly decreased by the injection of ophthalmic viscoelastic, as BH150 + V (*n* = 14; -10.92 ± 4.09%—*p* = 1.000) showed a cell loss comparable to the control group.

**Conclusions:**

ECL is altered by I/A BH and reduced when viscoelastic substances are used.

## What is known

- Cataract surgery induces corneal endothelial cell loss. Several factors, such as ultrasound energy or lens fragments colliding with the endothelium, contribute to corneal endothelial cell damage. However, the effects of the bottle height during irrigation and aspiration without phacoemulsification have not been investigated so far.

## What this paper adds

- A higher irrigation bottle height induces increased endothelial cell loss independent from phacoemulsification.
- Ophthalmic viscoelastic substances serve as an endothelial protective agent against endothelial cell damage.

## Introduction

Cataract surgery (CS) causes significant corneal endothelial cell loss (ECL) [1–5]. The protection of the corneal endothelium is of critical importance in all patients undergoing intraocular surgery but needs to be respected even more in patients with low endothelial cell density (ECD) or very dense cataracts, and thus bear the risk of postoperative endothelial decompensation. Among several factors causing cell damage, such as ultrasound energy, also irrigation and aspiration (I/A) is discussed to alter surgery-related ECL [6–8].

The term “phacodynamics” refers to external adjustable factors related to the operation of phacoemulsification machines, such as ultrasound energy and fluidics. Fluidics describe the combination of in- and outflow mainly regulated by irrigation and aspiration (I/A), which is important with regards to several procedural aspects, especially to ensure consistent operating conditions, such as maintaining a stable and well-pressurized anterior eye chamber (AC) during phacoemulsification and to facilitate intraocular lens injection. In passive systems, inflow is mainly regulated by changing the bottle height (BH) of the irrigation fluid. Hydrostatic pressure, depending on the BH, prevents the AC from collapsing. With increasing BH, intraocular pressure (IOP) increases, causing deepening and stabilization of the anterior eye chamber, which improves operating conditions. The fluid flow rate is controlled by aspiration (vacuum) and fluid outflow. An imbalance of in- and outflow will lead to either hypotony with anterior chamber shallowing or hypertonia with increased stress on intraocular tissue. It appears reasonable that BH should be just as high as necessary, however, currently, there is no existing recommendation on the optimal BH to be used during I/A in cataract surgery to minimize ECL. In clinical studies no dense cataracts are used due to standardization and therefore ECL is often marginal. Consequently, different treatment regimens often do not show any significant effect between the investigated groups in clinical studies. Furthermore, cataract surgery related ECL is multifactorial, so that only slightly damaging single factors may multiply in a real-life setting.

Therefore, flow rate and IOP vary depending on the individual surgeon’s preferences with unclear consequences for the ECD. So far, it is unclear whether fluid turbulences during cataract surgery or temporary IOP peaks account for increased ECL. In order to establish a more endothelial protective surgery technique, this study aimed to investigate the sole influence of I/A applied via a phacoemulsification tip with a varying BH between 100 and 150 cm above eye level as well as the protective effects of an additional AC injection of a dispersive viscoelastic substance.

## Materials and methods

Split corneal buttons obtained from porcine eyes were used to elucidate whether I/A BH induces significant ECL. Following I/A at different BHs without phacoemulsification ECL was quantified over a period of 15 days as described previously [9–11].

### Globe preparation

Whole Porcine eyes from 6 ± 1-month old pigs were obtained from the local slaughterhouse. The eye bulbs were disinfected in a 1:20 iodine-PBS solution after orbital adnexes were removed within 12 h after slaughter. After disinfection, all eyes were irrigated in PBS and treatment was performed (controls were left untreated). For treatment and preparation, the eyes were positioned with the help of a bulb holder. Following I/A all eyes of the control and interventional groups were prepared to split corneal buttons (described below). in a laminar air flow unit and placed in culture medium within 10 h postmortem [9].

### Irrigation and aspiration

**Fig. 1:**
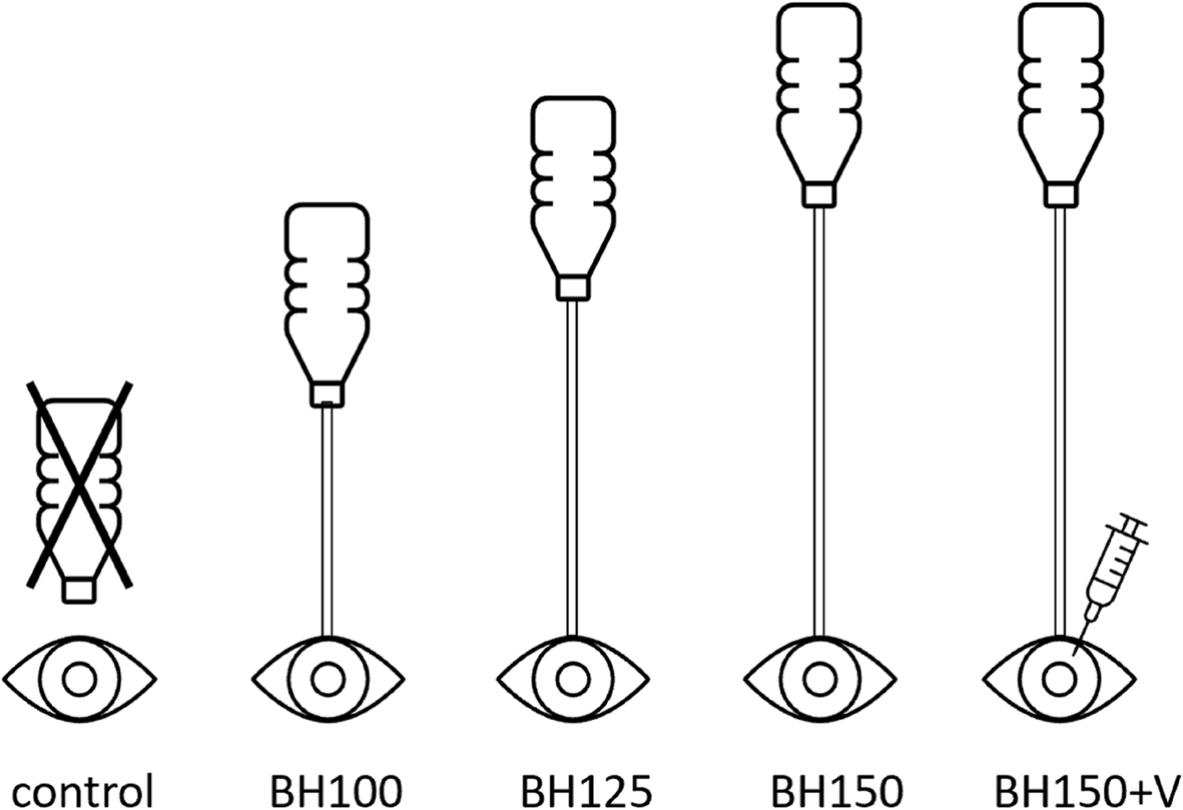
Schematic illustration of the experimental setup – five groups were compared in terms of I/A-induced corneal endothelial cell damage. Bottle height (BH) was varied between groups between 100 to 150 cm (BH100, BH125, BH150). Viscoelastic was used in one group to determine protective capabilities (BH150 + V)

For I/A a 30° phacoemulsification tip was inserted in bevel-down position into the AC through a 2.75 mm tunnel incision made with an ophthalmic slit knife (Mani, Utsonomiya, Japan) [11]. The phaco tip with I/A function was pushed forward horizontally towards the pupil’s center. The openings of the phaco sleeve were constantly directed sidewards. Five groups were formed to determine corneal endothelial cell damage possibly caused by I/A (compare Fig. 1):

- **Control** (no I/A; *n* = 13);
- **BH100** (I/A, BH 100 cm above eye level; *n* = 12);
- **BH125** (I/A, BH 125 cm above eye level; *n* = 14);
- **BH150** (I/A, BH 150 cm above eye level; *n* = 13);
- **BH150 + V** (I/A + dispersive viscoelastic, BH 150 cm above eye level; *n* = 14).

The PENTASYS phacoemulsification machine (Fritz Ruck GmbH, Germany) was connected to the phaco tip, settings were set to ultrasound energy 0%, irrigation 100–150 cmH_2_O, aspiration pressure 200 mmHg. Irrigation pressure varied passively according to differing BHs (BH – 100 cm; 125 cm; 150 cm). The given BH represents the difference in height between the eye and the fluid level in the fluid line. Balanced salt solution was used as irrigation fluid. In the intervention group BH150 + V, the AC was completely filled by a single injection of a dispersive ophthalmic viscoelastic (Opticel 2% (Hydroxypropyl methylcellulose (2% HPMC)), Ophthalmo Pro GmbH, Germany) prior to I/A for endothelial protection. I/A was performed for 10 min in the groups at the above-mentioned bottle heights. I/A was turned on and off every 10 s (30 cycles in total) equaling a total I/A time of five minutes.

### Split corneal buttons

After I/A, the porcine corneas were prepared to split corneal buttons as described previously [9]: a trephine with a diameter of 7.5 mm was used to partially trephine the cornea (epithelium, parts of the stroma). Lamellar dissection of the outer corneal layers was performed at the level of the trephined depth before the cornea was trephined in the remaining depth. Then, these split corneal buttons were transferred to culture medium (culture medium I (Biochrom GmbH, Berlin) supplemented with 2.5% fetal calf serum) and cultivated for 15 days at standard cultivation conditions (37 °C, 5% CO_2_) [9]. To ensure stable conditions during cultivation the culture medium was renewed on day 8.

### Cell count and endothelial cell staining

To assess corneal ECD on day 1 and 8, the split corneal buttons were placed in hypotonic balanced salt solution (hBSS: per 100 ml H_2_O: NaCl 490 mg; KCl 75 mg; CaCl × 2 H_2_O, MgCl × 6 H_2_O 46 mg, sodium acetate × 3 H_2_O 390 mg; sodium citrate x H_2_O 170 mg) to induce swelling of the cells. Then, the subjects were observed with a phase-contrast microscope (Nicon Eclipse Ti phase-contrast microscope) and ECD was assessed on pictures taken with the mounted camera. Digital microscopic photographs of the endothelium taken at 200x-magnification were analyzed using the EAS program (Endothelium analysis software, Robin Solutions, Germany) by semiautomated cell identification with manual correction on each of three 0.01 mm^2^-sized representative areas per cornea – then, mean cell counts were extrapolated to the dimensional unit “cells per mm^2^”. Three Pictures were taken from areas in the central cornea in each of the split corneal buttons. On day 15, the split corneal buttons were stained with 0.25% trypan blue (Merck, Germany) and 0.2% alizarin red S (Merck, Germany) [12], and again ECD was determined. Each specimen was assigned a three-digit number prior to cultivation for blinded assessment of cell density, which was then unmasked after the final cell count on day 15.

### Statistics

Statistical analysis to evaluate significance levels in ECD within and between groups was performed using ANOVA and pairwise dependent t-tests adjusted with Bonferroni method. The assumption of normality of residuals from linear model was tested with Kolmogorov test and the homogeneity of variances of residuals with Levene test. Both assumptions were met (*p* > 0.05). Differences in relative and absolute ECL between groups at day 15 follow-up were estimated via mixed-regression modeling technique with consecutive pairwise comparisons. The assumptions of the mixed-regression model were met as well. The resultant *p*-values were adjusted with Bonferroni method. Data is expressed as mean ± standard deviation (SD) (± 95%-confidence interval (CI)). Statistical significance was considered at *p* ≤ 0.05. All analyses were performed with R Core Team (2019) [13].

## Results

**Fig. 2:**
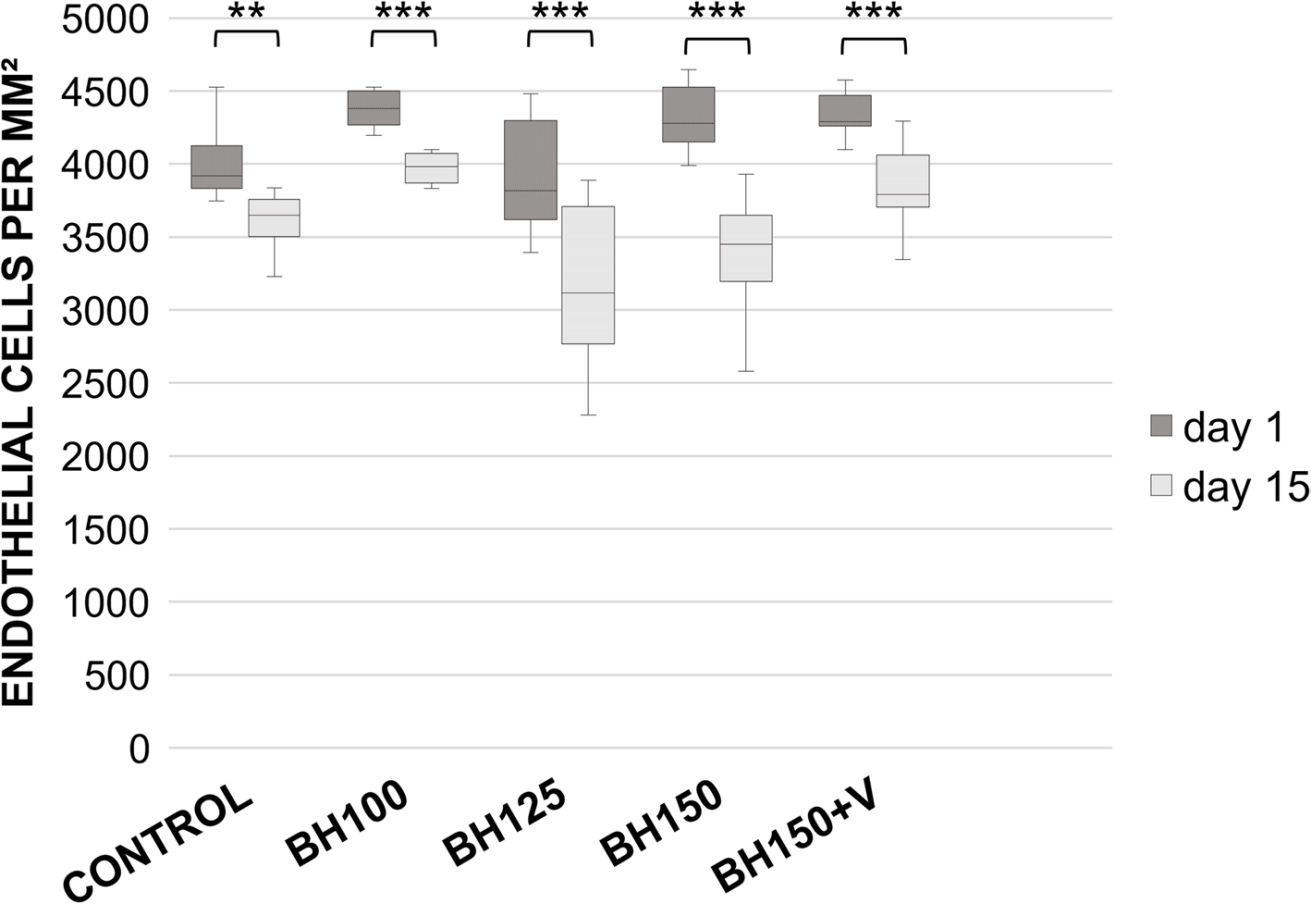
Box plots with whiskers providing endothelial cell densities of all groups on day 1 and on day 15. All groups showed significant endothelial cell losses after two weeks of cultivation. ECL was higher in BH125 and BH150 compared to the control group, BH100 and BH150 + V (also compare Fig. 3). Levels of significance: **p ≤ 0.01, ***p ≤ 0.001

**Fig. 3:**
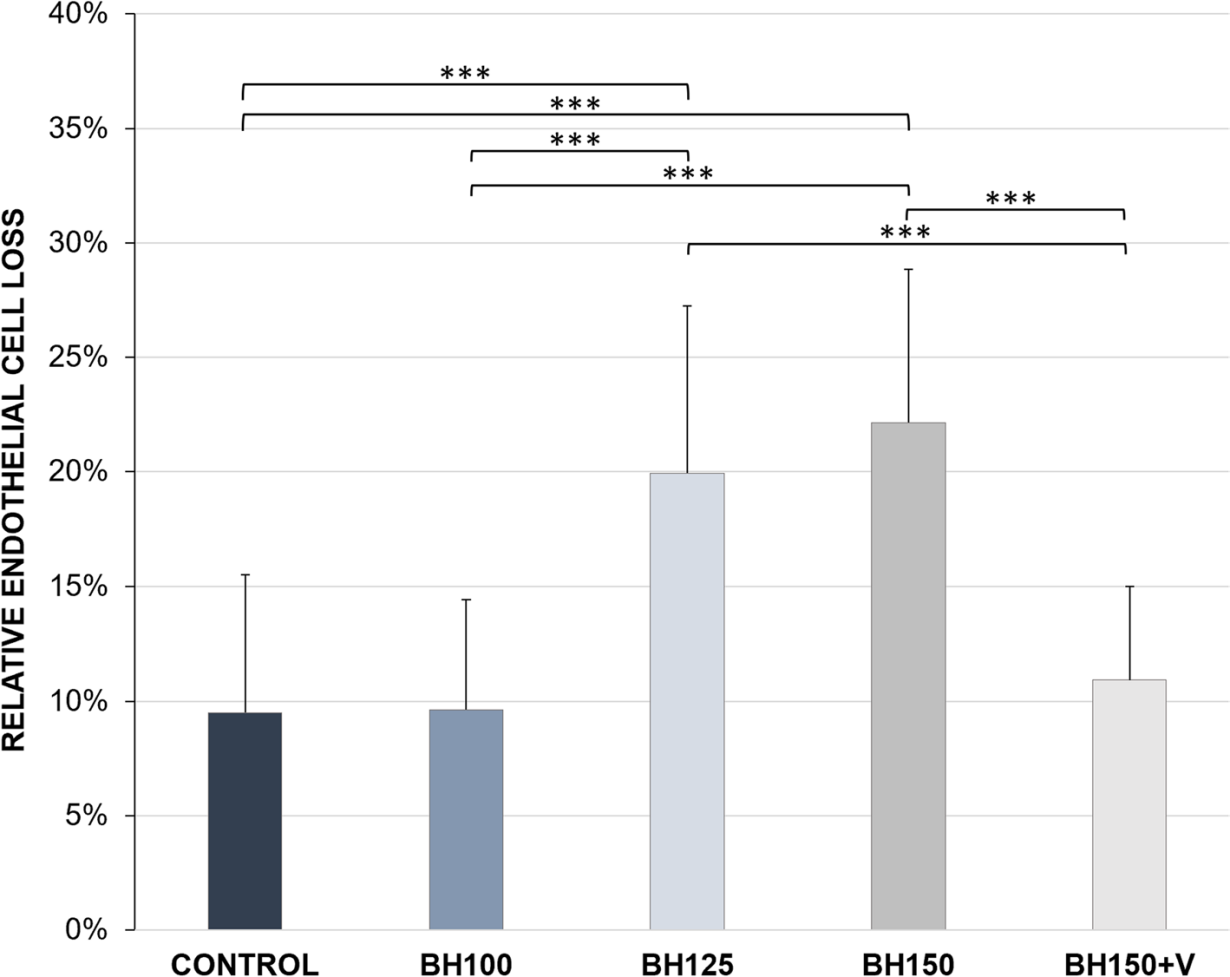
The relative endothelial cell loss (ECL) 15 days after irrigation/aspiration at different bottle heights is shown. See Table 1 for descriptive data. Data depicted as mean ± SD. Level of high significance at ***p < 0.001

A well-preserved continuous endothelial cell layer was found in all of the examinations on day 1, 8 and 15. The absolute ECD in the control group (3999 ± 230 (± 125) cells/mm^2^ (average ± SD (± 95%-CI))) and BH125 (3909 ± 379 (± 199) cells/mm^2^) was significantly lower (*p* < 0.05) on day 1 compared to the other groups, but all groups showed a regular ECD between 3909 ± 379 (± 199) cells/mm^2^ and 4356 ± 300 (± 170) cells/mm^2^ (see Table 1). Significant ECL (*p* < 0.05) was found in all groups after 15 days of cultivation (see Fig. 2). To allow adequate comparability, relative ECL (percentage of ECL day 15 compared to day 1) was calculated for each group (see Table 1 & Fig. 3). The control group showed a relative ECL of -9.69 ± 6.03 (± 3.16) %. While in the group BH100 a relative ECL of -9.69 ± 4.81 (± 2.52) % was not significant (*p* = 1.000) when compared to the control group, the groups BH125 (-19.44 ± 7.30 (± 3.82) %) and even more BH150 (-21.99 ± 6.70 (± 3.51) %) both suffered a highly significant increased relative ECL (*p* < 0.001). Thus, the relative ECL compared to the control group significantly increased from a BH of 125 cm onwards (see Table 1 and Fig. 3). The higher ECL in BH150 compared to BH125 did not reach level of significance (*p* = 1.000). By an additional injection of a dispersive viscoelastic prior to I/A into the anterior eye chamber the relative ECL was mitigated to -10.92 ± 4.09 (± 2.14) %, which corresponds to an ECL, that was not statistically significant higher (*p* = 1.000) compared to the control group (see Table 1 and Fig. 3). ECL in BH125 and BH150 was also significantly higher compared to BH100 and BH150 + V (BH100 vs. BH125 *p* < 0.001; BH100 vs. BH150 *p* < 0.001; BH150 + V vs. BH125 *p* = 0.004; BH150 + V vs. BH150 *p* < 0.001).

**Table 1:** Absolute and relative endothelial cell loss

	**Day 1** (cells/mm^2^)	**Day 14** (cells/mm^2^)	**Absolute ECL** (cells/mm^2^)	**Relative ECL** (%)	***p*-value on relative ECL (cell loss compared to control)**
Control	3999 ± 230	3611 ± 195	-388 ± 250	-9.69 ± 6.03	-
BH100	4356 ± 300	3934 ± 312	-422 ± 214	-9.69 ± 4.81	1.000
BH125	3909 ± 379	3149 ± 549	-760 ± 227	-19.44 ± 7.30	< 0.001
BH150	4324 ± 241	3373 ± 403	-951 ± 261	-21.99 ± 6.70	< 0.001
BH150 + V	4319 ± 188	3848 ± 258	-471 ± 172	-10.92 ± 4.09	1.000

In the two groups BH125 and BH150 endothelial cell loss (ECL) after two weeks of cultivation was markedly increased compared to control (and also compared to BH100 and BH150 + V). Thus, all groups suffered significant endothelial cell loss compared to day 1, whereas cell loss was significantly increased in BH 125 and BH150. *P* values refer to group comparison of the relative ECL with the control group after two weeks of cultivation. Data shown as mean ± SD. Significance was considered at *p* ≤ 0.05

**Fig. 4:**
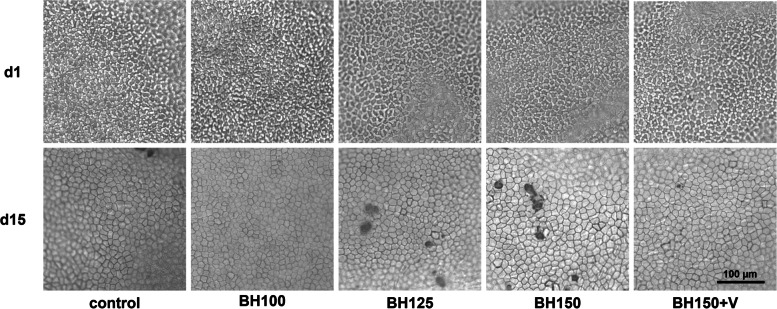
Representative photographs of corneal endothelial cells – Endothelial cells were photographed on day 1 (unstained) and day 15 (stained with trypan blue and alizarin red S). Significant endothelial cell decline was observed in BH125 and BH150 compared to control, whereas BH100 and BH150 + V did not. Enlargement of endothelial cells and damaged areas are clearly visible in BH125 and BH150. Scale bar corresponds to 100 µm

The control group showed an absolute ECL of -388 ± 250 (± 140) cells/mm^2^ or -9.69 ± 6.03 (± 3.16) % over a cultivation period of 15 days, which is similar to previous studies using this organ culture model and therefore is considered as the baseline ECL rate during the cultivation period of 15 days [9, 11]. Representative photographs of the endothelial cell layer of each group are shown in Fig. 4.

## Discussion

This is the first study investigating the sole influence of I/A without phacoemulsification using a standardized research model. So far, it was not clear whether I/A-related ECL is induced by I/A-related factors themselves, such as fluid turbulences or IOP increase, or by lens fragments colliding with the corneal endothelium [6–8, 14, 15]. We observed significant ECL caused by I/A, which correlated with a higher BH. Our data show, that I/A itself induces significant ECL at an irrigation BH of 125 cm and higher if I/A is applied for a total time of five minutes (30 on–off cycles in 10 min). The threshold for an increased ECL can be postulated to be somewhere between 100 to 125 cmH_2_O in our model, as the relative ECL after I/A at a BH of 100 cm was comparable to the control group (*p* = 1.000). These results can be interpreted independently from any influence of phacoemulsification and are consistent with our previously published study on enucleated porcine eyes on ultrasound energy simulating cataract surgery at an aspiration pressure of 200 mmHg and a BH of 105 cm, which also found no significant ECL as in this present study at an irrigation BH of 100 cm [11]. Evidently, a lower BH is favorable in terms corneal endothelial cell protection.

There are three relevant factors that have potentially contributed to I/A-induced ECL seen in our experiments:

First of all, low fluidic parameters seem to improve ECL [8]. In this context, also the the duration of I/A might be important, as longer irrigation time could apply more stress on the corneal endothelium. In our experimental setup, I/A time was identical in all I/A groups to rule out differing I/A times contributing to our results.

Secondly, I/A is necessary to maintain a certain IOP for adequate surgical conditions. The IOP increases with rising bottle height by raising hydrostatic pressure and has been discussed ambiguously to cause corneal endothelial cell loss in several studies, for example with regard to elevated IOP in primary open angle glaucoma [16–18]. However, it is assumed that ECL in glaucoma is more likely a result of moderate and long term elevated IOP levels and/or local antiglaucomatous medication—both factors are not present when looking at I/A-induced ECL. Shorter and more severe IOP peaks are seen in acute angle closure attack and were shown to cause significant ECL [19, 20]. An experimental study on rats demonstrated that a continuously elevated IOP of up to 83 mmHg for two hours induced endothelial cell loss [21]. Similar effects could therefore be expected during cataract surgery. As we report a cell loss rate of about 20% after I/A at a BH at 125 cm and 150 cm and 15 days of cultivation we question that the much shorter IOP peaks in our experimental model are the main source of ECL and other factors (e.g. fluidic turbulences) could rather contribute to ECL. Clearly, IOP increases with higher BH. Nonetheless, we have seen that the use of viscoelastic reduces ECL in the group BH150 + V. The present IOP in both groups with an identical BH (BH150 and BH150 + V) is assumed to be similar. The fact, that we performed periodic I/A cycles of 10 s as well as the peri-incisional leakage around the phaco tip certainly has lowered the IOP to some extent. Overall, an elevated IOP was not present for longer than a maximum of five minutes only, which is much shorter than in acute angle closure attack. To our knowledge, likewise there is also no clear evidence that short IOP peaks during cataract surgery are contributing to ECL. Still, it cannot be ruled out that an elevated IOP level induces some kind of stress on EC, which does not result in ECL primarily, but might influence ECL in combination with an additional stress factor. For example could endothelial cells be stressed by increased IOP (e.g. weakened cell–cell interactions) and then rinsed off or destroyed by irrigation turbulences. Unfortunately, IOP could not be measured in the research model used; however, in our opinion, IOP does not appear to be the responsible factor for the observed ECL.

The third possible factor are fluidic turbulences induced by repetitive I/A. With each start of irrigation, the turbulence in the anterior chamber increases. The hydrostatic pressure increases with a greater BH, and so does the pressure gradient leading to stronger fluidic turbulences. It seems highly probable that increased turbulences exert stress on the endothelial cell monolayer.

Other negative factors that could potentially do harm to the corneal endothelium are excluded in the experimental model (ultrasound phacoemulsification, swirling lens fragments colliding with the endothelium, etc.). The study by Suzuki et al. (2009) also investigated the effect of I/A with two different BH on ECL, but in contrast additionally performed phacoemulsification, which adds numerous variables (ultrasound energy (phaco time, phaco energy), distance from phaco tip to endothelium, different cataract grades, etc.) to the system with unclear side effects on the corneal endothelium and limits a reliable assessment of the influence of I/A itself. The study concludes that ECL is caused by increased IOP levels, but neglects to discuss a possible impact of fluidic turbulences during I/A [14]. A recent study by Ungricht et. al (2021) aimed to quantify corneal endothelial cell damage in rabbit eyes caused by irrigation flow with two different volumes (250 mL and 500 mL) at a fixed irrigation flow rate without varying BH and found no significant differences in endothelial cell damage [22]. A higher volume at the same fixed irrigation rate results in a longer irrigation time with non-variating turbulences. Also, most likely turbulences in the study by Ungricht et al. (2021) were very low, because a continuous flow at a fixed flow rate results in a rather laminar flow type, whereas we induced turbulent flow with switching I/A on and off every 10 s, which means that a flow from “no flow” to “maximum flow” was induced repetitively for 10 s every cycle. Some resilience of the corneal endothelium to irrigation can be expected, which is also confirmed in our study. Unfortunately, Ungricht et al. (2021) only quantified endothelial cell damage immediately after the irrigation procedure, which possibly masks a prolonged cell death rate, that we could observe in our study after two weeks of corneal cultivation. A study by Abouali et al. (2011) presented a computational model showing how the intensity of turbulence kinetic energy, that emerges from the phacoemulsification tip in the AC, increases with higher flow rates, and thus stronger turbulences with potential negative effects on ECL are to be expected [23]. However, in most clinical studies on this topic published so far, fluidic turbulences did not result in measurable ECL. A prospective randomized clinical trial evaluated the effect of low (aspiration flow rate (25 cc/min; BH 70 cm and 90 cm; vacuum ≤ 400 mmHg) and high (aspiration flow rate 40 cc/min, BH 90 cm and 100 cm; vacuum ≤ 650 mmHg) fluidic parameters during cataract surgery [8]. Also here, no significant differences in the rate of change in ECD three months postoperatively were observed between the two groups (low: -4.67 ± 0.15% vs. high: -5.22 ± 2.84% (*p* = 0.45)) [8]. Interestingly, AC flare, AC cells and corneal clarity was significantly better in the group with the low fluidic parameters [8]. An Iranian publication from 2009 also reported no significant effect on ECL between low (vacuum 200 mmHg, flow rate 20 cc/min) and high vacuum (vacuum 400 mmHg, flow rate 40 cc/min) phacoemulsification with a maximum BH of 100 cm [6]. Yet another study analyzing the impact of low (20 cc/min; BH 70 cm; vacuum 400 mmHg) and high (35 cc/min; BH 70 cm; vacuum 500 mmHg) fluidic settings on ECL, similarly did not reveal statistical significant differences (low: 4.92 ± 10.94% vs. high: 6.26 ± 15.48%; *p* = 0.696) [7]. All cited studies had in common, that viscoelastic substances were injected prior to phacoemulsification and BH never exceeded 110 cm. Therefore, all these studies are compatible with our results. Interestingly, the study with the highest analyzed BH of 110 cm, observed significant results concerning AC flare, AC cells and corneal clarity showing that a higher BH results in increased stress of intraocular tissues [8]. Unfortunately, there is no clear statement on the applied I/A time in above mentioned studies, which compromises the comparability to our study.

In general, the ECL in studies on cataract surgeries are often very low (approximately 5% ECL), which makes it very difficult to obtain significant results [8, 24–26]. Vice versa, a study focusing on dense cataracts, where the ECL is critically increased, would result in an inhomogeneous cohort as the applied ultrasound energy would differ between each patient outweighing the accuracy of analyzing the effects of the fluidic parameters. Consequently, the standardization of all ECL influencing factors (ultrasound energy, swirling lens fragments, duration of cataract surgery) seems nearly impossible in the clinical setting – especially in dense cataracts. Moreover, if several unfavorable factors are combined, their impact on corneal endothelial integrity may potentiate (for example stronger impact of lens fragment onto the corneal endothelium due to stronger turbulences). Due to the fact that phacoemulsification was conducted in all three of the above mentioned studies, the exclusive effects of I/A on ECL cannot be entirely quantified and interpreted independently from ECL induced by ultrasound energy or swirling lens fragments [6–8]. The effect of fluidic parameters on ECL can only be analyzed in detail in an experimental setting excluding all remaining involved factors, which is a main strength of our study.

Among the limitations of this study is the fact that pig eyes, not human eyes, were used for the investigations. However, human corneas are needed for corneal transplantation and experimental studies simulating cataract surgery using a higher number of human eyes are not practicable. Our research model based on fresh, unboiled porcine eyes has shown comparable ECL rates during cultivation as human donor corneas [9–11, 27]. The eyes were processed within ten hours after death, which is an acceptable time frame, especially when compared to cornea banking, where corneas are allowed to be harvested and cultivated up to 72 h after death in Germany. This is further underlined studies that have found no difference in endothelial cell density, primary graft failure or infection rate between corneas that were cultivated within < 12–14 h or > 12–14 h after death [28, 29]. The experimental setup was chosen to induce possible ECL to reveal further details on the influence of I/A BH on ECL. Nonetheless, our experimental workflow with intermittent periods of I/A is therefore artificial and total I/A time and flow peaks could be shorter in real-life cataract surgery. Also, the results of this study need to be seen with the limitation that in-vivo ECL possibly caused by I/A may behave differently compared to the loss rates observed in culture medium. ECD preoperatively could not be determined in our research model, but as the relative and not absolute ECL rates were determined postoperatively, the different treatment groups can be compared to each other. Furthermore, the chosen aspiration pressure of 200 mmHg was low compared to the clinical situation. With a higher aspiration pressure though, the AC was not stable anymore at a BH of 100 cm [11]. Consequently, the fluidic turbulences and the induced ECL might increase with higher aspiration pressure. To analyze the effect of fluidic turbulences on ECL at higher aspiration pressure settings further investigations are necessary.

Conclusively, ECL correlates with increased irrigation BH of more than 100 cm. Ophthalmic viscoelastic substances serve as protective agent against I/A-induced ECL. To our knowledge, this is the first study investigating the sole influence of I/A without simultaneous phacoemulsification on corneal ECL. Further studies are needed to investigate how ECL is altered by I/A including phacoemulsification with unmodified and standardized phaco settings but with varying bottle heights.

## Data Availability

The datasets used and/or analysed during the current study are available from the corresponding author on reasonable request.

## Abbreviations

AC: Anterior chamber
BH: Bottle height
ECL: Endothelial cell loss
IOP: Intraocular pressure
I/A: Irrigation and aspiration
